# Direct interaction between the hepatitis B virus core and envelope proteins analyzed in a cellular context

**DOI:** 10.1038/s41598-019-52824-z

**Published:** 2019-11-07

**Authors:** Florentin Pastor, Charline Herrscher, Romuald Patient, Sebastien Eymieux, Alain Moreau, Julien Burlaud-Gaillard, Florian Seigneuret, Hugues de Rocquigny, Philippe Roingeard, Christophe Hourioux

**Affiliations:** 1 INSERM U1259 MAVIVH – University of Tours and CHRU of Tours, Tours, France; 2 Plate-Forme IBiSA des Microscopies, PPF ASB – University of Tours and CHRU of Tours, Tours, France

**Keywords:** Cellular microbiology, Hepatitis B virus

## Abstract

Hepatitis B virus (HBV) production requires intricate interactions between the envelope and core proteins. Analyses of mutants of these proteins have made it possible to map regions involved in the formation and secretion of virions. Tests of binding between core and envelope peptides have also been performed in cell-free conditions, to study the interactions potentially underlying these mechanisms. We investigated the residues essential for core-envelope interaction in a cellular context in more detail, by transiently producing mutant or wild-type L, S, or core proteins separately or in combination, in Huh7 cells. The colocalization and interaction of these proteins were studied by confocal microscopy and co-immunoprecipitation, respectively. The L protein was shown to constitute a molecular platform for the recruitment of S and core proteins in a perinuclear environment. Several core amino acids were found to be essential for direct interaction with L, including residue Y132, known to be crucial for capsid formation, and residues L60, L95, K96 and I126. Our results confirm the key role of L in the tripartite core-S-L interaction and identify the residues involved in direct core-L interaction. This model may be valuable for studies of the potential of drugs to inhibit HBV core-envelope interaction.

## Introduction

Despite the availability of a vaccine for preventing hepatitis B virus (HBV) infection, this virus remains a major public health problem, as the 260 million people with chronic HBV infection worldwide have a high risk of major liver complications, such as cirrhosis and hepatocellular carcinoma^1^. Current treatments for HBV infection are essentially based on nucleos(t)ide analogs and immune modulators, such as pegylated-interferon, which inhibit viral replication and virus production, but the virus is only rarely completely cleared. Viral relapse occurs almost systematically when treatment is stopped, highlighting the need for additional therapies^2^.

HBV is an enveloped DNA virus of the *Hepadnaviridae* family. The oligomerization of its core protein (HBc) generates an icosahedral capsid approximately 34 nm in diameter, containing a relaxed circular (rc) partially double-stranded (ds) DNA genome of 3.2 kb^3^. The capsid is processed in association with reverse transcription^4^ and becomes enveloped through budding into a host-derived lipid bilayer membrane harboring the viral envelope proteins, leading to secretion of the mature virion^5^. Two types of non-infectious particles are also secreted: genome-free envelope capsids, also known as empty particles^6^, and subviral envelope particles (SVPs)^7,8^. Several hypotheses have been proposed to explain the secretion of mature and empty particles, but not of immature particles. These hypotheses include structural modifications of the core protein^4,9^ and the presence of single-stranded (ss) DNA or pre-genomic (pg) RNA in assembled core constituting a signal blocking the envelopment of immature particles^5,6^.

The core protein has three domains: (i) the 140 amino-acid (aa) N-terminal domain (NTD), mostly structured into an alpha-helical domain known to be involved in capsid assembly^10,11^; (ii) a linker formed by residues 141–149, of unknown function but potentially involved in the regulation of capsid assembly^12^; and (iii) the basic, arginine-rich C-terminal domain (CTD) formed by residues 150–183, involved in viral genome packaging through its interaction with a complex of pgRNA and polymerase^13^. The three dimensional (3D) structures of the NTD and the full-length core have been determined by X-ray diffraction and cryoelectron microscopy^11,14,15^. They contain five alpha helices, including the α3 and α4 helices forming a protuberance at the capsid surface, called the spike, which is involved in core dimerization. The fifth helix and the downstream loop are involved in dimer oligomerization. The site of interaction with the envelope proteins, the matrix-binding domain (MBD), is thought to lie in the core spikes^16,17^, but remains poorly characterized. Several residues exposed at the surface of the capsid were identified by mutagenesis as potentially involved in these interactions with the ability of these mutants to form nucleocapsids and secreted virions^18^. Eleven of the 52 residues tested blocked virion secretion, but had no effect on nucleocapsid assembly. These residues are located in diverse regions of the protein, suggesting that structural details of the entire core protein are important for virion secretion.

The HBV envelope consists of three closely related envelope proteins: small (S), middle (M) and large (L), all of which have identical C-terminal ends. These proteins self-assemble to form non-infectious SVPs, which are produced in a 10^3^- to 10^6^-fold excess over infectious virions^5,19^. The S protein is necessary and sufficient for SVP formation and also essential for HBV morphogenesis^7^. The M protein, containing an additional preS2 domain, is not required for either HBV morphogenesis or infectivity^20^. Finally, the L protein, which contains the additional preS1 domain and has two types of transmembrane topology (e-preS *versus* i-preS), is essential for two steps of the viral cycle^21^. In its e-preS conformation, the preS1 region of the L protein is exposed at the surface of the virion and interacts with the viral receptor at the hepatocyte membrane^22–25^. In its i-preS conformation, the preS1 region is involved in interactions with the capsid via a short conserved domain, the matrix domain (MD), which has been mapped to the preS1/preS2 junction^23,26,27^.

The interplay between core and envelope proteins for the production of infectious or empty particles was previously studied by genetic studies^18,26,28,29^. In addition, the use of synthetic peptides showed *in vitro* that the preS1-preS2 junction was required to interact with patient-derived or recombinant HBV core particles^30,31^. In this study, we investigated HBV envelope-core interactions further, by assessing the ability of a set of envelope and core protein mutants to bind to each other in a cellular context. These core and envelope mutants were selected from studies of Bruss *et al*. (1997) and Ponsel *et al*. (2003) who showed their importance in the production of infectious particles. Using confocal imaging and biochemical approaches, we demonstrated an interaction between the L and core proteins, leading to the preferential localization of core within the cytoplasm. The S protein did not interact directly with the core protein, but the L protein served as a molecular platform, recruiting the S and core proteins to the same subcellular environment at the periphery of the nucleus. We found that a lack of capsid formation resulted in an absence of L-core interaction, and that, conversely, capsid formation was not sufficient for the recognition of L by core. These results shed light on the early events in HBV morphogenesis, paving the way for characterization of the mechanisms by which highly potent compounds targeting the core protein can inhibit the replication cycle.

## Results

### Wild-type, tagged and mutant S, L and core proteins

**Figure 1:**
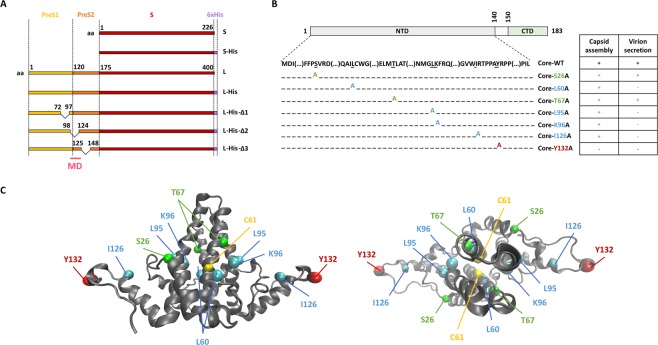
Diagram of the S, L and core proteins and their mutated derivatives. (**A)** The WT S envelope protein consists of the S domain (red) and the WT L envelope protein consists of this domain together with two other domains at its N-terminus: the preS1 (yellow) and the preS2 (orange) domains. The matrix domain (MD) is indicated (pink). Deletions are indicated by a bridge linking the flanking residues. Some constructs contain a His-tag at their C-terminus (purple box). **(B)** The WT core protein consists of the NTD domain (light gray), a linker (white) and the CTD domain (green). Seven mutant core proteins were generated by alanine substitution. The ability of mutated core proteins to assemble and to produce virions, according to the findings of Ponsel *et al*.^18^, is summarized in the table on the right. The names of the constructs are shown in color, according to their capsid assembly and virion secretion properties: C+V+ residues in green, C+V− residues in blue, C-V- residues in red. **(C)** Orthogonal views of the 3D structure of the core NTD dimer linked by C61 residues (in yellow). This structure was obtained by crystallography (#1QGT)^11^. The backbone is represented as a gray ribbon. Mutated residues are shown in the CPK model, with colors as in the table in (**B**).

We used the WT sequences of the S and L proteins. We also added a His-tag to the C-terminal end of these sequences, to take into account the cross-reactivity of the anti-HBs antibody (Fig. 1A). Three mutant forms of the L protein were designed, in which 20–25 amino acids were deleted from the pre-S1 region, the junction between the pre-S1 and pre-S2 regions or the pre-S2 region, generating the L-His-Δ1, L-His-Δ2 and L-His-Δ3 mutants, respectively (Fig. 1A).

Residues of the core protein identified in previous studies as affecting various phases of virion morphogenesis, such as capsid assembly and envelope incorporation, were replaced by alanine residues^18^. A synthetic table of these substitutions and their effects, as reported in previous studies, is shown in Fig. 1B, and their localization in the dimer of the core protein structure is presented in Fig. 1C.

### Analysis of the production of wild-type, tagged and untagged envelope and core proteins

**Figure 2:**
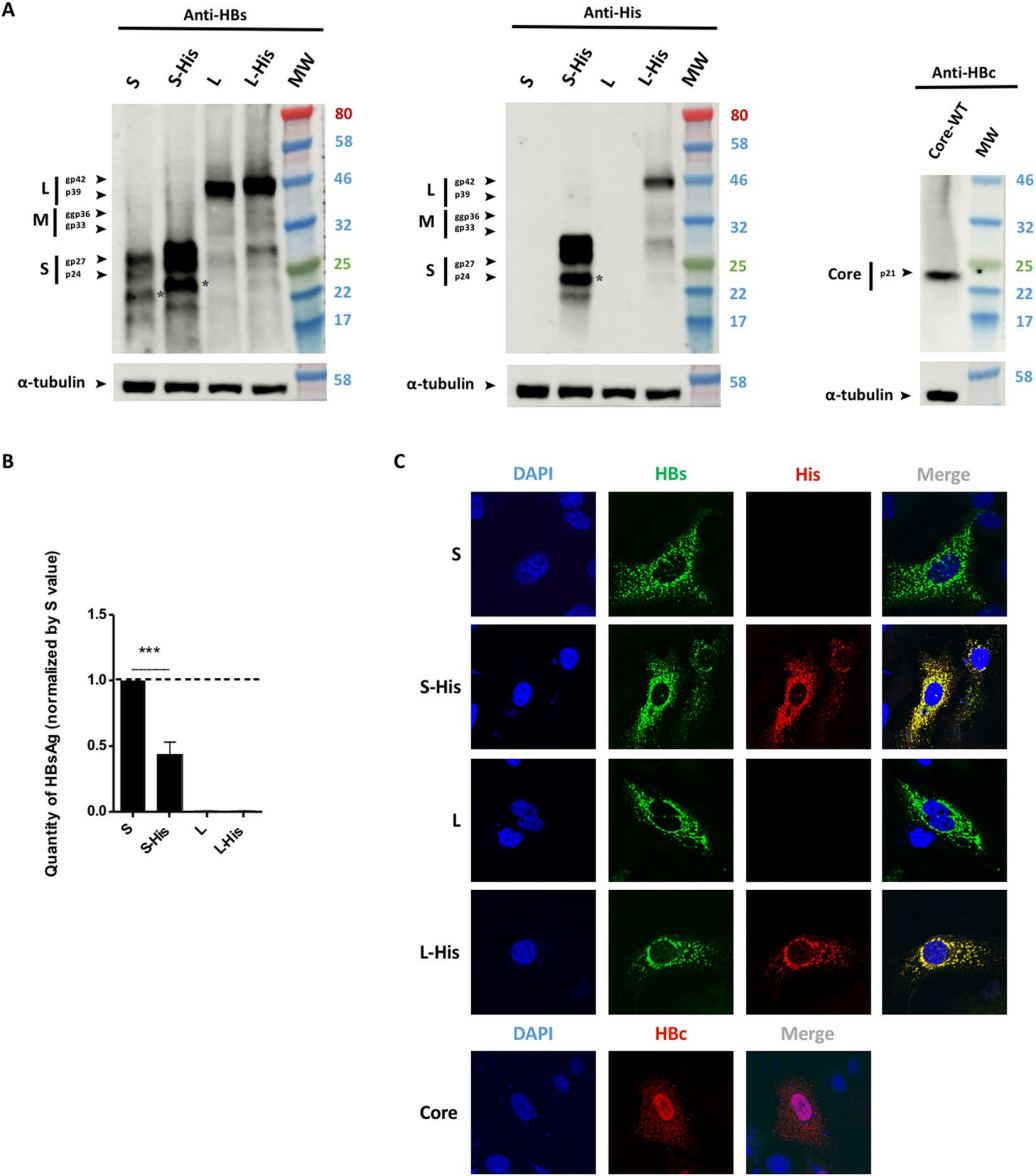
Envelope and core protein levels. Huh7 cells were transfected with a plasmid encoding the untagged WT HBV S protein or S-His, or WT untagged L, L-His or core proteins. Three days after transfection, the cells were analyzed by western blotting for HBsAg secretion, and by confocal microscopy. **(A)** Cell lysates were separated by SDS-PAGE, the bands were transferred onto membranes and the membranes were probed with anti-HBs (left panel), anti-His (central panel) or anti-HBc (right panel) antibodies. The asterisk (*) notes the presence of a truncated version of the S and S-His proteins. **(B)** A commercial ELISA was used to quantify HBsAg in cell supernatants. The amount of S-His secreted was about half the amount of WT S secreted. The bars indicate the mean ± standard deviation (SD) values from four independent experiments. *p* values (paired *t*-tests) were determined: ***p value < 0.001. **(C)** Cells were fixed on coverslips and proteins were visualized by confocal microscopy after indirect immunofluorescence with an anti-HBs antibody (in green) together with an anti-His antibody (in red) for the L or S proteins, or with an anti-HBc antibody (in red) for the core protein. Nuclei were labeled with DAPI (in blue).

We first produced the WT S and L proteins and their His-tagged homologs in Huh7 cells, to evaluate the impact of the tag sequence added to their C-terminal ends of these proteins. Immunoblots on cell lysates performed three days after transfection showed that both the WT and tagged forms of S and L were specifically detected by an anti-HBs antibody (Fig. 2A, left panel). The S-His and L-His envelope proteins detected displayed a slight shift in molecular weight relative to the untagged proteins, consistent with the insertion of the tag sequence. S and S-His proteins were revealed by two close bands at 24 and 27 kDa and by a lighter single band at 22 kDa. Samples were treated by the N-glycosylase to demonstrate that the two higher bands correspond to the co-migration of glycosylated and non-glycosylated S protein. The lighter band was also sensitive to N-glycosylase and may correspond to a truncated form of the S protein (Fig. S1). Interestingly, the glycosylation of the S-His showed that His-tag had no impact on the membrane topology of the envelope protein. As expected, an internal ATG initiation site within the L sequence enabled to initiate production of low amounts of M and S proteins. An immunoblot performed with an anti-His antibody showed the specific detection of these tagged proteins at their expected size, with no signal recorded for their untagged forms (Fig. 2A, central panel).

We then assessed the secretion of these proteins into the cell culture medium. HBsAg quantification in cell supernatants showed that cells producing the tagged S protein secreted about half as many SVPs as cells producing the untagged S protein (Fig. 2B). This finding is consistent with the higher intracellular levels of S-His than of WT S observed on the anti-HBs immunoblot in Fig. 2A (left panel). The accumulation of S-His within cells is, thus, correlated with lower levels of secretion for this protein, consistent with previous studies showing an effect of S modification on SVP secretion^32,33^. Nevertheless, our S-His protein was able to form secreted SVPs, albeit in smaller amounts. By contrast, as expected, L protein production did not lead to SVP secretion into the supernatant, whether or not the protein was tagged (Fig. 2B), and there was therefore no imbalance in the amounts of protein detected on the immunoblot (Fig. 2A).

We next compared the cellular distributions of unlabeled and His-tagged proteins by confocal microscopy with anti-HBs and anti-His antibodies. The untagged S and L proteins yielded similar reticular patterns of fluorescence, mostly in the perinuclear area, consistent with the insertion of these proteins into endoplasmic reticulum membranes^8^ (Fig. 2C, first and third rows). The absence of a fluorescence signal with the anti-His antibody demonstrates the specificity of the anti-His antibody for labeled envelope proteins. The detection of the tagged proteins with both anti-HBs and anti-His antibodies revealed a perfect superimposition of fluorescence (Fig. 2C, second and fourth rows), with an *r* value of 0.80 ± 0.03 for S-His and 0.87 ± 0.03 for L-His. We used this experiment to set up internal controls for our calculated *r* giving high scores for both His-tagged proteins.

We also checked core production. A single specific 21 kDa band was detected on immunoblots (Fig. 2A, right panel). The confocal imaging of cells producing core protein and immunolabeled with an anti-HBc antibody revealed a strong fluorescent signal, mostly located in the nucleus, although some diffuse fluorescence was also detected in the cytoplasm (Fig. 2C).

### L interacts with the core protein whereas S does not

**Figure 3:**
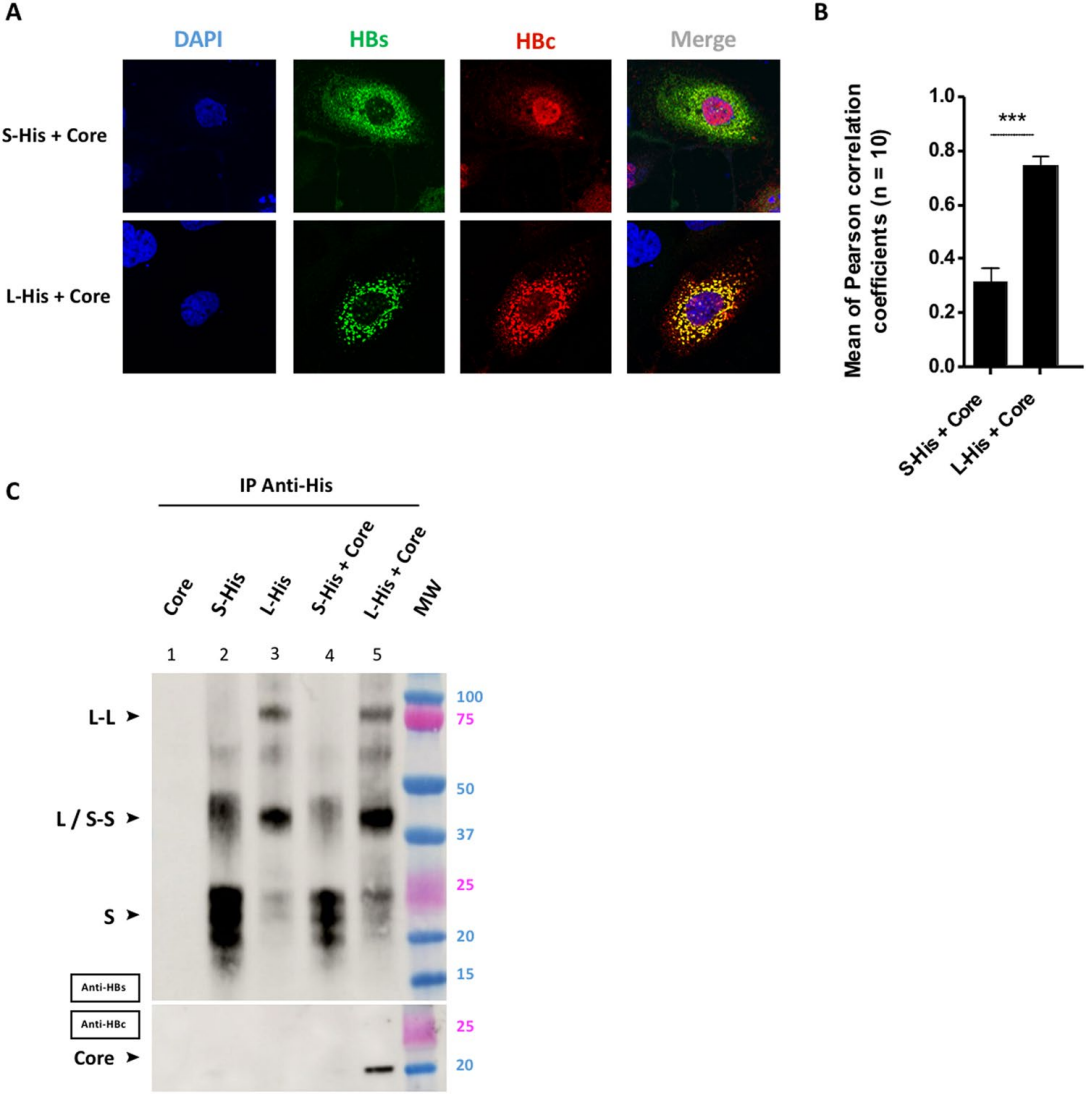
Only the L protein interacts with the core protein. Huh7 cells were cotransfected by incubation for three days with a plasmid encoding the S-His or L-His protein together with a plasmid encoding the core protein. **(A)** Huh7 cells were fixed and double-stained with anti-HBs (in green) and anti-HBc (in red) antibodies, and the nuclei were counterstained with DAPI (in blue). Each image corresponds to the major phenotype observed. Note that the colocalization of envelope and core proteins was observed only for the L-His and core proteins. **(B)** Histogram of *r* values. Differences between L-His/Core and S-His/Core colocalizations were analyzed statistically by Mann-Whitney tests on 30 images from 3 different experiments (***p < 0.001). **(C)** Huh7 cell lysates were collected and 400 µg of total protein was subjected to IP with an anti-His antibody. Membranes were probed with an anti-HBs (IP) or an anti-HBc (Co-IP) antibody. Note that the co-IP of core protein was observed only in the presence of L-His protein.

The colocalization of the envelope and core proteins was analyzed by immuno-fluorescence staining, and their interactions were analyzed by co-immunoprecipitation (co-IP; Fig. 3). Three days after cotransfection, considerable differences in the subcellular distribution of core were observed, according to the co-expressed envelope protein (Fig. 3A). When co-expressed with S-His, core was detected mostly in the nucleus, as in cells expressing core alone (Fig. 2C), and was weakly co-localized with S-His (Fig. 3A, upper row). An *r* value for S-His and core colocalization of 0.32 ± 0.1 (Fig. 3B) was obtained on image analysis, consistent with the slight yellowish color of the cytoplasm. By contrast, when co-expressed with L-His, core protein was no longer detected in the nucleus, instead displaying clear cytoplasmic sequestration (Fig. 3A, lower row). The strong superimposition of the green and red channels in the merged image resulted in an intense yellow color and a high *r* value of 0.75 ± 0.03 (Fig. 3B).

The resolution of optical fluorescence microscopy is insufficient to demonstrate an interaction between two proteins. We therefore performed specific co-IP experiments with an antibody against the C-terminal His-tag of the envelope proteins. We first confirmed the specificity of the anti-His antibody for the immunoprecipitation of proteins harboring the His-tag, by demonstrating an absence of core precipitation (Fig. 3C, well 1). When IP experiments were performed on cell lysates expressing S-His or L-His (Fig. 3C, wells 2 and 3), bands were detected corresponding to the expected masses of 20–25 kDa and 40 kDa for S-His and L-His, respectively. Higher molecular weight bands detected at 40 kDa and 80 kDa may also have corresponded to S-S and L-L dimers, respectively (Fig. 3C, wells 2 and 3). In experiments on cell lysates co-expressing the S-His or L-His and core proteins, the core protein was found to be co-immunoprecipitated with the L-His protein (Fig. 3C, well 5), but not with the S-His protein (Fig. 3C, well 4). This result demonstrates the existence of a strong interaction between L-His and core proteins, but not between S-His and core proteins. These data are consistent with the confocal microscopy images demonstrating a colocalization of the L-His and core proteins and the absence of colocalization between S-His and core proteins (Fig. 3A).

### The L envelope protein acts as a bridge between the core and S proteins in the cytoplasm

**Figure 4:**
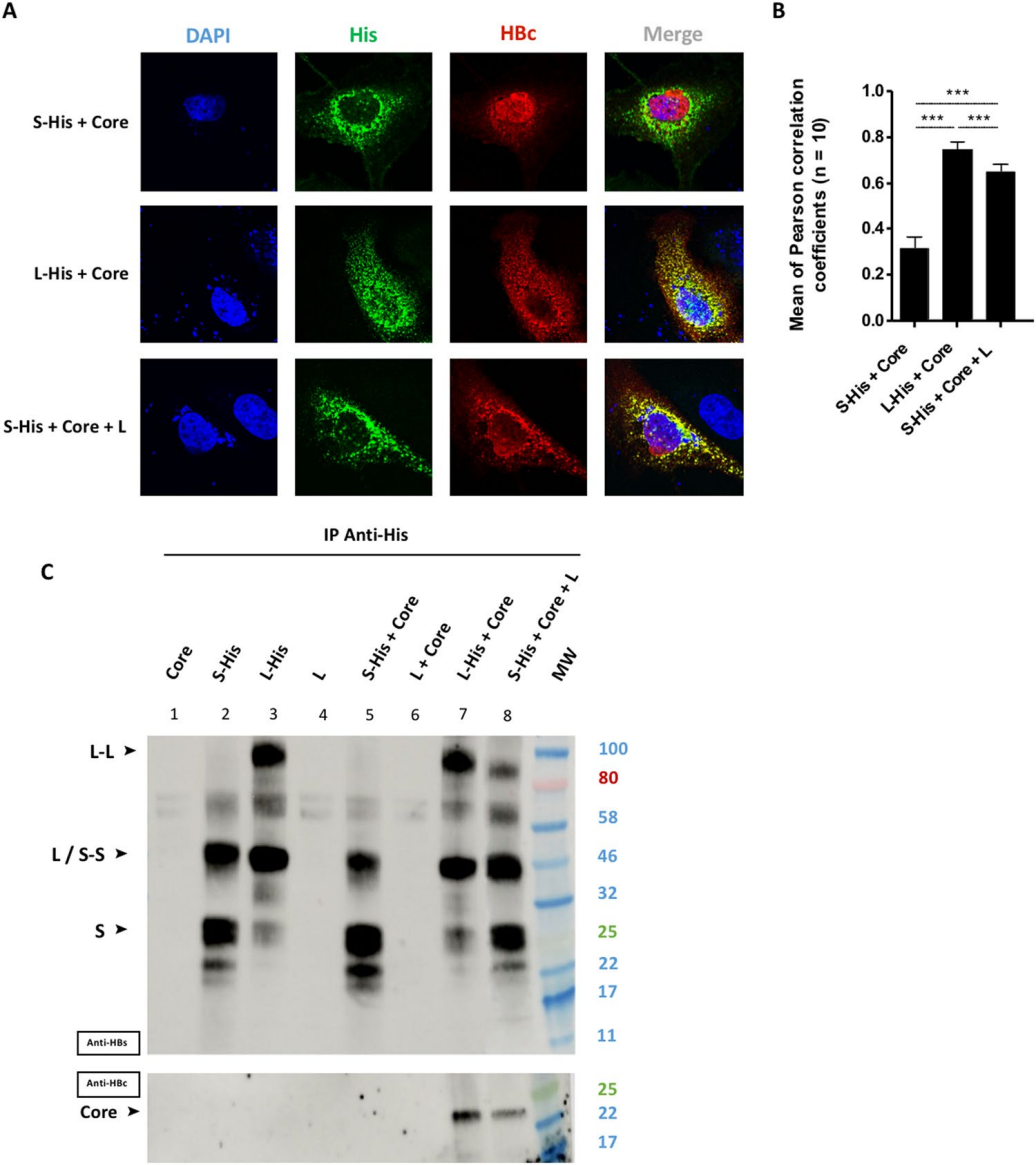
Tripartite interaction between the L, S and core proteins. Huh7 cells were cotransfected with a plasmid encoding the core protein, and a plasmid encoding S-His, or L-His, or S-His plus untagged L. Three days post-transfection, cells were analyzed by confocal microscopy and co-IP. **(A)** Cells were fixed and double-stained with anti-His (in green) and anti-HBc (in red) antibodies, and nuclei were labeled with DAPI (in blue). S-His was colocalized with WT core protein in the presence of untagged L proteins (bottom row). **(B)** Histogram of *r* values (***p value < 0.001). **(C)** Huh7 cell lysates were collected and 400 µg of total protein was subjected to IP with an anti-His antibody. The immunoprecipitated samples were blotted and the membranes were probed with anti-HBs (IP) or anti-HBc (Co-IP) antibodies. Core protein co-IP was observed with S-His if untagged L was also produced.

The S protein is essential for the formation and secretion of virions and SVPs^19,34,35^. We therefore investigated whether the heterodimerization of L and S proteins^36^ favored the recruitment of the S protein at the site of the L-core complex. We compared the distributions of the S and core proteins in the presence of the L protein (Fig. 4). We avoided the cross-detection of the two envelope proteins by the anti-HBs antibody by co-expressing the untagged L protein with the S-His protein. We first confirmed the localization of the core protein for each envelope protein, demonstrating, as expected, an absence of co-localization between S-His and core (Fig. 4A, first row and Fig. 4B), and a strongly colocalization of the L-His and core proteins (Fig. 4A, second row and Fig. 4B). However, strong colocalization was observed between the S-His and core proteins in the presence of the untagged L protein (Fig. 4A, third row and Fig. 4B, *r* = 0.64 ± 0.03). This result suggests that the L protein recruits both S and core proteins in the cytoplasm. To investigate whether such tripartite interaction occurs in HBV replicating cells, the pRVHBV1.5Δenv plasmid encoding for a replicative HBV genome, defective for the expression of envelope proteins was cotransfected with two plasmids expressing L-His and S-Flag. Interestingly, the same colocalisation of the three partners was observed in this replicative context (Fig. S3).

We investigated the possibility of a direct interaction between the three proteins, by immunoprecipitating S-His or L-His from cell lysates expressing various combinations of L, S and core proteins. Immunoblot membranes were probed with anti-HBs (Fig. 4C, upper panel) or with anti-HBc (Fig. 4C, lower panel) antibodies, to monitor IP and co-IP, respectively. Staining with anti-HBs antibody (upper membrane) revealed a large number of bands, which were identified by comparison with Fig. 3C. The specificity of the anti-His antibody for IP was demonstrated by the immunoprecipitation of proteins harboring the His-tag but not of unlabeled proteins (Fig. 4C, wells 1, 4 and 6). We also confirmed the co-IP of the core protein with L-His (Fig. 4C, well 7), but not with S-His (Fig. 4C, well 5). The principal finding of this experiment (Fig. 4) was the IP of S-His accompanied by the detection of both the untagged L (upper membrane) and core proteins (lower membrane) (Fig. 4C, well 8). This pool down of L and core proteins was detected specifically with the S-His protein, as neither of these proteins was detected in absence of S-His (Fig. 4C, well 6). Slightly less core protein was immunoprecipitated than with the L-His and core proteins alone (compare Fig. 4C, wells 7 and 8), confirming the significant decrease in *r* factor in similar situations (Fig. 4B). These results suggest that the S, L and core proteins are assembled into a tripartite complex, in which L plays a key role in recruiting both the S and core proteins.

### The preS1-preS2 junction domain of the L envelope protein mediates interaction with the core protein

As the L protein seemed to play a key role in core recruitment, we investigated the domains of these two proteins required for their interaction. We used the L-His-Δ1, L-His-Δ2 and L-His-Δ3 mutants carrying deletions in the preS1 and/or preS2 domains (fully described in Fig. 1A) to address this question for the L protein. All mutant proteins were produced and validated in the conditions developed for the L proteins. Comparisons of immunoblots and confocal images with those for the L-His protein revealed no significant differences, with similar levels and subcellular distributions for all mutant proteins (Fig. S2).

**Figure 5:**
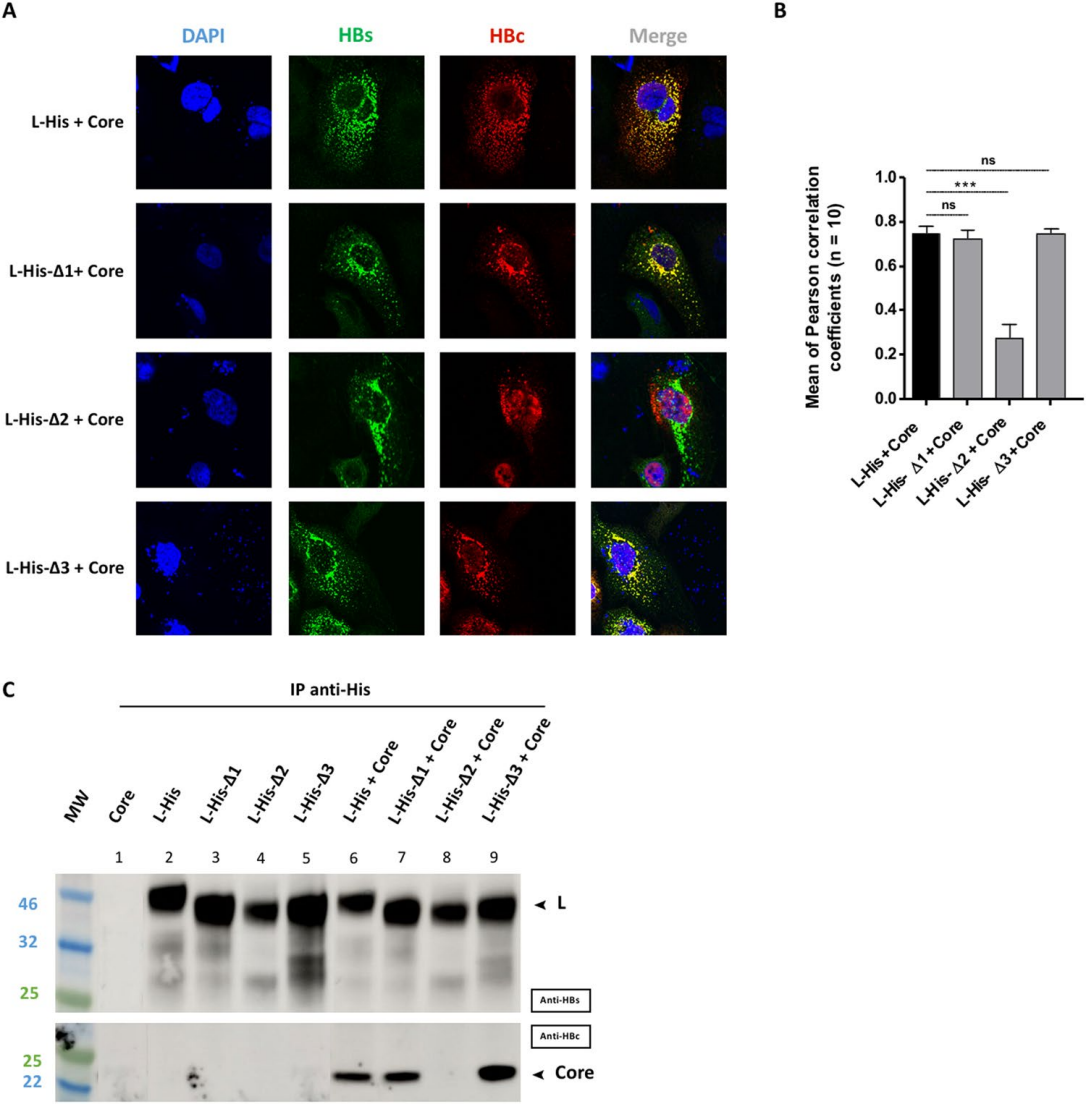
Interaction between the mutant L proteins and the core protein. Huh7 cells were cotransfected with plasmids encoding the L-His, L-His-Δ1, L-His-Δ2 or L-His-Δ3 protein and a plasmid encoding the core protein. Three days post-transfection, cells were analyzed by confocal microscopy and co-IP. **(A)** Cells were fixed and double-stained with anti-HBs (in green) and anti-HBc (in red) antibodies, and nuclei were labeled with DAPI (in blue). Perfect colocalization with the core protein was observed for the L-His protein, and the L-His-Δ1 and L-His-Δ3 mutant proteins, but not for the L-His-Δ2 mutant. **(B)** Histogram of *r* values as previously described (***p value < 0.001). **(C)** Huh7 cell lysates were collected and 400 µg of total protein was subjected to IP with an anti-His antibody. Membranes were probed with anti-HBs (IP) or anti-HBc (Co-IP) antibodies. Well 1 is a control, demonstrating a lack of core protein immunoprecipitation with the anti-His antibody. Wells 2–5 are controls showing that L-His and L-His derivatives are immunoprecipitated with the anti-His antibody. Core protein co-IP was observed with L-His and all derivatives except for the L-His-Δ2 mutant.

We then investigated the ability of these mutant L proteins to recruit the core protein. Confocal imaging revealed a strong colocalization of the L-His-Δ1 and L-His-Δ3 mutants with the core protein (Fig. 5A, second and fourth rows, and Fig. 5B), as for the L-His protein, used as positive control (Fig. 5A, first row and Fig. 5B). By contrast, the co-expression of the L-His-Δ2 mutant with core protein resulted in a very weak colocalization of these two proteins (Fig. 5A, third row), as confirmed by the low coefficient obtained (0.27 ± 0.06, Fig. 5B) and the visualization of the core protein within the nucleus. Co-IP experiments yielded results consistent with the confocal imaging findings (Fig. 5C). The core protein was clearly coprecipitated with the L-His protein, and the L-His-Δ1 and L-His-Δ3 mutants (Fig. 5C, wells 6,7 and 9 respectively), but not with the L-His-Δ2 mutant (Fig. 5C, well 8), suggesting that the Δ2 deletion in the preS1-preS2 junction is involved in the interaction of L with the core protein.

### The L protein interacts with the MBD region of the core protein

We also performed similar experiments with core mutants in which key residues in the matrix-binding domain (MBD) were replaced with alanine residues (fully described in Fig. 1). The cellular distribution and expression level of each mutant were first checked by confocal imaging and western blotting, respectively (Fig. S4). With the exception of the core-Y132A mutant, which was predominantly located in the cytoplasm, all the mutants had subcellular distributions similar to that of the WT core protein: mostly nuclear but with a diffuse cytoplasmic signal (Fig. S4,A). Western blot analysis revealed that all mutants were produced in similar amounts to the WT core protein (Fig. S4,B).

**Figure 6:**
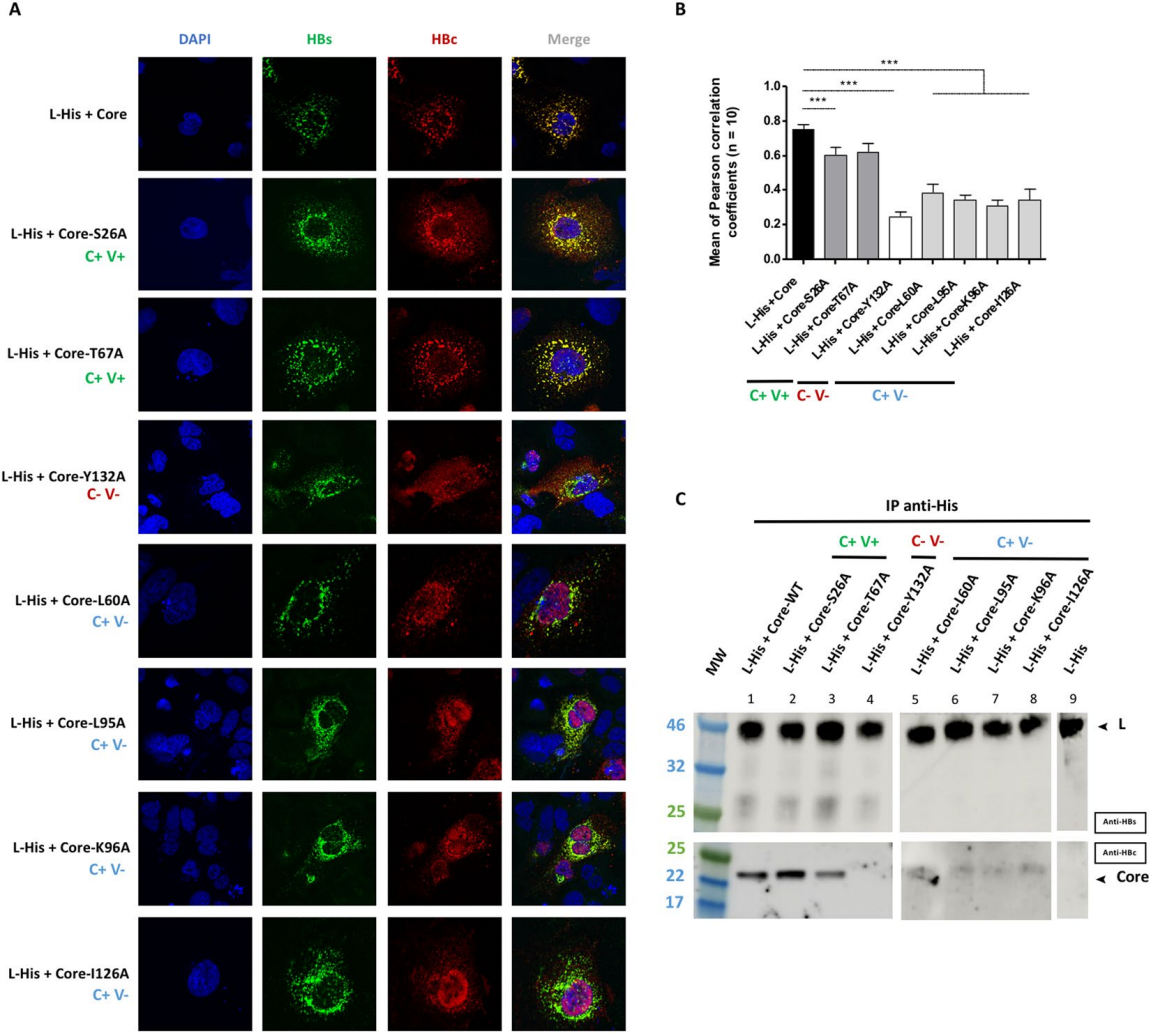
Interaction between the L protein and the mutant core proteins. Huh7 cells were cotransfected with plasmids encoding the L-His protein and the WT core protein or one of the seven mutant core proteins. Three days post-transfection, cells were analyzed by immunolabeling and confocal microscopy, and co-IP was investigated with L-His as the bait. **(A)** Cells were double-stained with anti-HBs (in green) and anti-HBc (in red) antibodies, and nuclei were labeled with DAPI (in blue). As for the WT controls, a strong colocalization of the L-His and core proteins was observed with C+V+ mutants (core-S26A and coreT67A). Partial colocalization between the L-His and core proteins was observed with C+V− mutants (core-L60A, core-L95A, core-K96A, core-I126A) and no colocalization of L-His and core proteins was observed for the C-V- mutant (core-Y132A). **(B)** Histogram of *r* values, as previously described (***p value < 0.001). The capsid formation and secretion properties of each mutant are shown at the bottom of the figure. **(C)** Huh7 cell lysates were collected and 400 µg of total protein was subjected to IP with an anti-His antibody. The immunoprecipitated samples were subjected to immunoblotting and the membranes were probed with anti-HBs (IP) or anti-HBc (Co-IP) antibodies. The capsid formation and virion secretion properties of each of the mutant proteins are reported at the top of the figure. The C+V+ mutants were strongly co-immunoprecipitated with the WT L-His. Weak but detectable co-IP was observed with the C+V− mutants, whereas no signal was obtained with the C−V− mutant, as for the controls (simple transfection of each partner) (data not shown). All the blot acquisitions were processed in parallel and correspond to the same experiment.

We then cotransfected cells with the L-His envelope protein and these mutant core proteins, for evaluations of colocalization by confocal microscopy (Fig. 6A) and of interaction by co-IP (Fig. 6B). The two C + V + mutants (Core-S26A and Core-T67A) retained the ability to colocalize with L-His protein, as shown by confocal imaging (Fig. 6A, compare the first row with the second and third rows) and confirmed by the high *r* value (0.60 ± 0.05 and 0.62 ± 0.05 respectively), similar to that for the WT core protein (0.75 ± 0.03) (Fig. 6B). These two mutants also interacted directly with the L-His protein in co-IP experiments (Fig. 6C, wells 2 and 3), like the WT core (Fig. 6C, wells 1). The C-V- mutant (Core-Y132A) was unable to colocalize with the L-His protein (Fig. 6A, fourth row), as confirmed by the very low Pearson’s coefficient (0.25 ± 0.03; Fig. 6B), or to interact with L-His, as shown by the absence of co-IP (Fig. 6C, well 4). All four C + V− mutants (Core-L60A, Core-L95A, Core-K96A and Core-I126A) displayed poor colocalization with WT L-His (Fig. 6A, the last four rows), with low *r* values (0.40 ± 0.05, 0.24 ± 0.03, 0.30 ± 0.04 and 0.34 ± 0.06, respectively). On co-IP, these four mutants gave a similar, very faint band (Fig. 6C, wells 5–8), indicating that they had largely lost their ability to interact directly with the L-His protein. Overall, these results indicate that residues L60, L95, K96, I126 and Y132, in the MBD of the core protein, are essential for interaction with the L protein.

## Discussion

During the egress of HBV particles, the capsid shell formed in the cell is enclosed by the viral envelope, which is composed of cellular lipids and viral envelope proteins, in the endoplasmic reticulum^5,8,37^. The production of infectious virions is also accompanied by the production of subviral particles (SVPs) enriched in S proteins, and empty viral particles^5^. The domains of the envelope and core proteins potentially required for capsid envelopment were mapped in previous molecular studies with mutants, in which virions secretion in the supernatant of transfected cells was used as the readout^18,26,38–41^. Core-envelope interaction was also studied in cell-free binding assays, with patient-derived or recombinant HBV capsids and envelope peptides^30,31^. We extended these studies by using confocal microscopy and biochemical approaches to investigate direct protein-protein interaction between HBV core and envelope in a cellular context. We first demonstrated that the L protein interacts with the core protein, leading to a redistribution of core, which becomes mostly cytoplasmic and barely detectable in the nucleus. The preS1-preS2 junction deletion was associated with a loss of L-mediated core relocalization and an absence of co-immunoprecipitation, consistent with the essential role of this sequence in the production of infectious particles^26,29^. A weak but detectable colocalization of the S and core proteins was observed on confocal microscopy, despite the absence of S-mediated core protein precipitation. This suggests that the interaction between the core and S proteins previously reported in cell-free binding assays and analyses of mutants in molecular genetic studies^27,30,31^ may be very weak. This finding is consistent with a recent study showing that the co-expression of HBV constructs and a plasmid expressing S resulted in only low levels of empty particle secretion^29^. The S protein may therefore be essential for the membrane curvature required for viral budding, rather than directly involved in capsid recruitment. Interestingly, an interaction between core and S was observed in the presence of L protein (Fig. 4C). This interaction was further confirmed in cells expressing the HBV genome and transcomplemented with tagged envelops (Fig. S3). The description of core-envelope interaction on cells expressing only our proteins of interest may thus accurately recapitulate the interplay between core and envelope proteins in HBV-replicating cells. Interestingly, domains for the homo- and likely hetero-oligomerization of S and L proteins were mapped in the transmembrane domain 2, the cytosolic and the luminal loops far from the preS1-preS2 junction suggesting that the L-S interacting domains may not overlap the L binding domain for the core protein. Thus, our findings suggest that the L protein may act as a molecular platform, recruiting the various viral components. Such a role would be consistent with the multifunctional nature of this protein, reflecting its unusual dual topology, with domains on the cytoplasmic and luminal sides of the membrane^21,32^. A careful analysis of the results presented in Fig. 4B,C revealed that the colocalization of S and core proteins in the presence of L protein was less efficient than the colocalization of L and core proteins (Fig. 4B, compare the second and third bars). This trend was also confirmed by the smaller amount of core protein immunoprecipitated by S in the presence of L than of core protein immunoprecipitated by L (Fig. 4C, compare wells 7 and 8). This may result from the co-expression of the L and S proteins restoring SVP formation and secretion, thereby decreasing the amount of local membrane L available for interaction with the core protein. However, this hypothesis requires confirmation in further studies.

Based on the structure of the NTD^18,28^, Bruss *et al*. introduced a series of point mutations in the core gene sequence, resulting in the substitution of alanine for various residues in the encoded protein. They then classified the resulting mutated proteins into groups on the basis of their ability to form a capsid and to produce virions in the cell supernatant. We used some of these mutants here, to study interactions with the L protein. A first group of two mutants, S26A and T67A, previously reported to form capsids and to produce virions^18^, gave results similar to those for the wild-type core protein. S26 is part of a flexible loop between helices 1 and 2 located far from the matrix-binding domain (MBD). T67 is in the upper part of the third helix, fully exposed to the solvent, and, like other residues in this helix, it does not seem to participate in dimer formation^11^. Thus, neither of these residues is involved in interactions with L via their side chains, and the mutations concerned probably have only a minor effect on global core structure.

A second group of mutants, L60A, L95A and K96A, gave intermediate results, highlighting the importance of the residues concerned for interactions between core and L (Fig. 6). These mutations have been shown to inhibit the production of mature viruses^18,28^ but not the production of empty particles^29^. The impact of these mutations on interactions between core and envelope proteins is clearly much greater than that on the production of empty virions described by Ning *et al*.^29^. This discrepancy may reflect the use of a plasmid containing the entire viral genome in this study, whereas our experiments were carried out with several different plasmids each encoding a protein of interest. Furthermore, the content of the cell medium was followed by native gel electrophoresis in the study by Ning *et al*., whereas the core-envelope interaction was followed by the L-mediated immunoprecipitation of core protein in stringent conditions. We thus suggest that the L60, L95 and K96 side chains may be involved in interactions with L though hydrophobic (L60 and L95) or hydrophilic (K96) links, and that point mutations affecting these side chains are probably sufficient to reduce core-envelope recognition. Alternatively, such mutations may interfere with a structural domain of the core protein. Indeed, despite the distance between L60 and the L95 and K96 residues, these amino acids all lie in the same area within the 3D structure. This area was recently identified as a hydrophobic pocket in the center of the spike^42^. Indeed, a study of the structure of an NTD-F97L mutant showed that this mutation had no major impact on the structure of the spike, instead causing only a small structural modification in the center of the two spikes. The authors suggested a direct role for this hydrophobic pocket in the core-dependent recruitment of L, with its modification decreasing the efficiency of particle envelopment during the budding process^43^. By analogy with this F97L mutant, it is tempting to speculate that the replacement of L60, L95 and K96 with shorter residues (and a neutral residue for K96) modified the hydrophobic pocket, abolishing envelope recognition. Meanwhile, a comparative decrease in L recognition was also observed when the I126 residue was replaced with the shorter, hydrophobic alanine residue (Fig. 6). This residue is located at the end of the fifth helix, and the mutagenesis of its neighbors, V124, W125 and R127, suggested that this region was essential for wild-type capsid assembly^44,45^, which, in turn, allows virion secretion^46^. Thus, the loss of interaction is probably correlated with an assembly defect of this mutant protein.

Finally, the high sensitivity of L for interaction with a native capsid structure was highlighted by the Y132A mutant. The Y132 residue is part of a proline-rich loop essential for dimer interaction and is fully buried in the capsid structure during subunit interaction^11^. The Y132A mutant has been extensively studied and is known to display an impairment of salt-induced homologous assembly^47^. Nevertheless, the Y132A mutant can form well-defined structures that are different from the native structures^15^. Thus, our results indicate that, even in the presence of dimers of Y132A mutants harboring all the residues required for L interaction, a native structure of for the core protein is essential for this interaction.

Interestingly, these previous studies by Bruss and coworkers were carried out on replication-competent particles containing the full HBV genome^18,28^. By contrast, we studied non-replicative particles lacking the viral genome. Nevertheless, a correlation between particle envelopment and direct interaction between the core and L proteins was established, despite the absence of capsid maturation. Our results thus suggest that core-envelope interaction is not dependent on a maturation signal driven by rcDNA formation or the CTD phosphorylation state, consistent with reports of the secretion of RNA-containing particles^48,49^ and a recent study showing that CTD phosphorylation does not significantly modify the structure of the capsid to generate a structural signal^42^.

We did not study complete virion morphogenesis here, but this approach nevertheless constitutes an attractive model for exploring interactions between the HBV core and envelope proteins. In addition, since the mutated residues are highly conserved in all HBV genotypes (Supplemental Table 1), our observations made with a genotype A virus can be extended to the other genotypes.

Given the need to improve treatments for HBV infection, we believe that such investigations could play an important role in the design of potential antiviral strategies. Drugs inhibiting envelope or core protein assembly have recently been developed and might be valuable in combination with current drugs targeting the replication of the viral genome, to achieve a synergistic blockade of the HBV viral cycle^13,50^. Similarly, improvements in our understanding of HBV core-envelope proteins interactions might help us to develop complementary approaches, such as peptide inhibiting core-envelope interaction, and our model might be useful for studies of their effects in a cellular context.

## Methods

### Plasmids

DNA sequences encoding the wild-type (WT) or mutant forms of the envelope or core proteins were inserted into pcDNA3.1(+) (Invitrogen, Invitrogen Corporation Pontoise, France). PCR amplification was performed with the pRVHBV1.5, a plasmid containing 1.5 copy of a genotype A2 HBV genome, as the template (kindly provided by Dr Volker Bruss, accession number: X02763), and specific primers carrying *Bam*HI and *Xho*I or *Afl*II and *Apa*I restriction sites. A 6xHis-tag (His-tag) was also inserted in phase at the C-terminal end of the L and S protein sequences. Finally, L-His-Δ1, L-His-Δ2 and L-His-Δ3 mutants carrying deletions in preS1-preS2 domains were generated by overlap amplification with specific dedicated primers, using the L sequence as a template (Fig. 1). Seven core protein mutants carrying unique amino-acid substitutions were designed: core-S26A, core-T67A, core-L60A, core-L95A, core-K96A, core-I126A and core-Y132A (Q5® Site-Directed Mutagenesis Kit, NEB). Constructs were checked by sequencing and expression in cells. The primers used for amplification and/or mutagenesis are summarized in Supplemental Table 2.

### Cell culture and transfection with plasmids

Huh7 cells were maintained at 37 °C in Dulbecco’s modified Eagle medium supplemented with 10% fetal calf serum (Gibco, France) and 1% antibiotic mixture (penicillin/streptomycin, Invitrogen Corporation Pontoise, France). Cells were used to seed six-wells plates containing a 12 mm coverslip, at a density of 2 × 10^5^ cells/well. Transfections were performed by incubating cells with 2 µg of plasmid and jetPEI^TM^ (Life Technologies, Saint Aubin, France) in accordance with the supplier’s recommendations.

### HBsAg quantification

The supernatants of transfected cells were harvested, filtered and diluted 1:100 for analysis in the commercial ARCHITECT HBsAg Qualitative II assay (Abbott Laboratories, Rungis, France). The results obtained in relative light units (RLU) were normalized with the value obtained for the WT S protein.

### Confocal microscopy, immunoblotting and co-immunoprecipitation

Three days post-transfection, the coverslips were removed from the wells and treated for confocal microscopy, and the remaining cells were lysed for co-immunoprecipitation and western-blot experiments.

For microscopy, cells were fixed by incubation for 15 min in 4% paraformaldehyde in PBS, permeabilized by incubation for 20 min with 0.05% saponin and saturated with 0.5% bovine serum albumin (BSA) in PBS. The cells were then stained with a 1:2000 dilution of human anti-HBc polyclonal antibody^51^, a 1:500 dilution of mouse anti-HBs monoclonal antibody (H25B10, Merck) or a 1:1000 dilution of rabbit anti-His polyclonal antibody (ab9108, Abcam). Cells were then washed in PBS for 15 min and incubated with the corresponding secondary antibody coupled to Alexa Fluor 488 or 594 (Thermo Fisher Scientific) diluted 1:2000. Coverslips were mounted in an aqueous anti-fade medium containing DAPI (Fluoromount-G^TM^, Thermo Fisher Scientific) and observed under a LEICA SP8 gSTED confocal microscope equipped with a 63x PL APO 1.40 CS2 oil-immersion objective. All images were recorded with standards settings, allowing to provide raw pictures. No one of these images was manipulated using an image processing software.

Colocalization was quantified as the percentage of proteins colocalized, with the “Squassh” plugin-in of ImageJ software, as previously described^52–54^. The Pearson’s coefficient of correlation (*r*) obtained (between −1 and +1) indicates the extent to which the two labels are colocalized. A mean ± SD (standard deviation) was obtained from 10 confocal images and *p* values (Mann-Whitney test) were obtained for comparisons.

The total resting cells were released from the wells by trypsin treatment and resuspended in ice-cold RIPA lysis buffer (50 mM Tris-HCl pH 7.4, 150 mM NaCl, 1% NP40, 0.15% sodium deoxycholate, 1 mM EDTA, 0.05% SDS) supplemented with a complete protease inhibitor cocktail from Roche (Meylan, France). The resulting lysate was centrifuged, and the total protein concentration of the supernatant was assessed with the Pierce BCA Protein Assay Kit (Thermo Fisher Scientific). We then denatured 50 µg of total protein by heating with 1% of β-mercaptoethanol-containing Laemmli loading buffer (Bio-Rad). This sample was then subjected to SDS-PAGE (8–16% acrylamide gel). The resulting bands were transferred onto a nitrocellulose membrane (Amersham, Orsay, France), and the membranes were blocked by incubation in 0.05% (v/v) Tween 20 (PBS-T) and 5% (wt/vol) non-fat milk powder in PBS and probed by overnight incubation at 4 °C with a 1:10000 dilution of human anti-HBc polyclonal antibody^51^, a 1:500 dilution of goat anti-HBs monoclonal antibody (70-HG15, Fitzgerald) or a 1:1000 dilution of rabbit anti-His polyclonal antibody (ab9108, Abcam). Protein levels were standardized against β-actin or α-tubulin, detected with a 1:2000 dilution of rabbit anti-β-actin antibody (ab8227, Abcam) or a 1:5000 dilution of mouse anti-α-tubulin antibody (ab56676, Abcam). Membranes were washed and incubated with horseradish peroxidase-labeled secondary antibody, which was then detected by enhanced chemiluminescence on an Imagequant LAS500 apparatus (GE Healthcare).

For immunoprecipitation, we incubated 100 µL of PBS-washed Sepharose beads (Rec-Protein G-Sepharose^TM^ 4B – Thermo Fisher Scientific) with 2 µg of goat anti-His monoclonal antibody (ab9108, Abcam) and 400 µg of clarified cell lysate overnight at 4 °C, with gentle shaking. The beads were washed with 0.2% Triton X-100 in PBS, the eluted proteins were analyzed by SDS-PAGE and blotting onto membranes, which were probed with a human anti-HBc polyclonal antibody^51^ (IP) or a goat anti-HBs monoclonal antibody (co-IP), as described above.

## Supplementary information

Figure_S1

Figure_S2

Figure_S3

Figure_S4

Supplemental_Table_1

Supplemental_Table_2

Supplemental_information_raw_data
