# Supplementary material for: Direct interaction between the hepatitis B virus core and envelope proteins analyzed in a cellular context
Source: Sci Rep. 2019 Nov 7;9:16178. doi: 10.1038/s41598-019-52824-z (PMC6838148; doi:10.1038/s41598-019-52824-z)
Supplement: Supplementary file 1 — Figure_S1 [file 41598_2019_52824_MOESM1_ESM.pdf]

Direct interaction between the hepatitis B virus core and envelope proteins analyzed in a cellular context

Florentin Pastor[1], Charline Herrscher[1], Romuald Patient[1], Sebastien Eymieux[1], Alain Moreau[1], Julien Burlaud-Gaillard[2], Florian Seigneuret[1], Hugues de Rocquigny[1,*], Philippe Roingeard[1,2*] and Christophe Hourioux[1,2,*]

[1]: INSERM U1259 MAVIVH – University of Tours and CHRU of Tours, Tours, France

[2]: Plate-Forme IBiSA des Microscopies, PPF ASB – University of Tours and CHRU of Tours, Tours, France.

To whom correspondence should be addressed:

[*]: hourioux@med.univ-tours.fr ; roingeard@med.univ-tours.fr; hderocquigny@univ-tours.fr

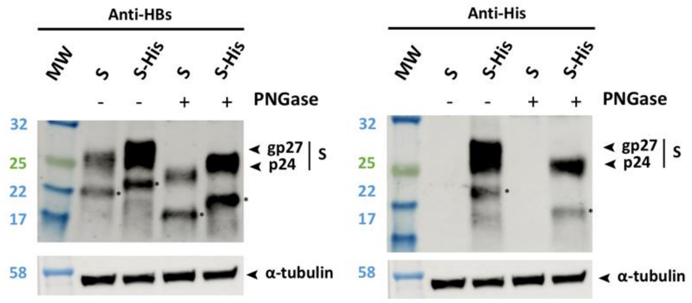

Figure S1. PNGase treatment of S and S-His proteins. Huh7 cells were transfected with a plasmid encoding the untagged WT HBV S protein or S-His. Three days after transfection, the cells were analyzed by western blotting. Cell lysates were treated or not by a N-glycosylase (PNGase) and then separated by SDS-PAGE. Membranes were probed with anti-HBs (left panel) and anti-His (right panel) antibodies. Protein normalization was controlled by α-tubulin. The asterisk (*) notes the presence of a truncated version of the S and S-His proteins which are also deglycosylated.
