# Supplementary material for: Direct interaction between the hepatitis B virus core and envelope proteins analyzed in a cellular context
Source: Sci Rep. 2019 Nov 7;9:16178. doi: 10.1038/s41598-019-52824-z (PMC6838148; doi:10.1038/s41598-019-52824-z)
Supplement: Supplementary file 2 — Figure_S2 [file 41598_2019_52824_MOESM2_ESM.pdf]

Direct interaction between the hepatitis B virus core and envelope proteins analyzed in a cellular context

Florentin Pastor[1], Charline Herrscher[1], Romuald Patient[1], Sebastien Eymieux[1], Alain Moreau[1], Julien Burlaud-Gaillard[2], Florian Seigneuret[1], Hugues de Rocquigny[1,*], Philippe Roingeard[1,2*] and Christophe Hourioux[1,2,*]

[1]: INSERM U1259 MAVIVH – University of Tours and CHRU of Tours, Tours, France

[2]: Plate-Forme IBiSA des Microscopies, PPF ASB – University of Tours and CHRU of Tours, Tours, France.

To whom correspondence should be addressed:

[*]: hourioux@med.univ-tours.fr ; roingeard@med.univ-tours.fr; hderocquigny@univ-tours.fr

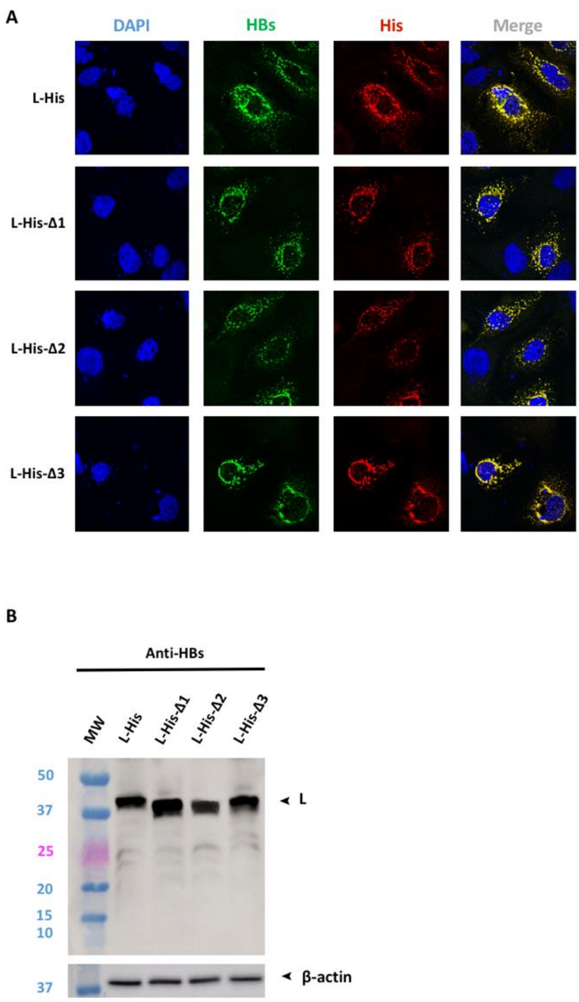

Figure S2. Expression of the mutant L proteins. Huh7 cells were transfected by inubation for three days with a plasmid encoding the L-His protein or the L-His-Δ1 or L-His-Δ2 or L-His-Δ3 mutant protein. **(A)** The subcellular distributions of the proteins encoded by all constructs were determined by confocal microscopy after double-staining with anti-HBs (in green) and anti-His (in red) antibodies. Nuclei were labeled with DAPI (in blue). Similar subcellular distributions were observed for all the mutant proteins. **(B)** Cell lysates were subjected to SDS-PAGE, and the resulting protein bands were blotted onto membranes, which were probed with anti-HBs antibody. The levels of the various proteins were compared with those of β-actin for quantification.
