# Supplementary material for: Direct interaction between the hepatitis B virus core and envelope proteins analyzed in a cellular context
Source: Sci Rep. 2019 Nov 7;9:16178. doi: 10.1038/s41598-019-52824-z (PMC6838148; doi:10.1038/s41598-019-52824-z)
Supplement: Supplementary file 3 — Figure_S3 [file 41598_2019_52824_MOESM3_ESM.pdf]

Direct interaction between the hepatitis B virus core and envelope proteins analyzed in a cellular context

Florentin Pastor[1], Charline Herrscher[1], Romuald Patient[1], Sebastien Eymieux[1], Alain Moreau[1], Julien Burlaud-Gaillard[2], Florian Seigneuret[1], Hugues de Rocquigny[1,*], Philippe Roingeard[1,2*] and Christophe Hourioux[1,2,*]

[1]: INSERM U1259 MAVIVH – University of Tours and CHRU of Tours, Tours, France

[2]: Plate-Forme IBiSA des Microscopies, PPF ASB – University of Tours and CHRU of Tours, Tours, France.

To whom correspondence should be addressed:

[*]: hourioux@med.univ-tours.fr ; roingeard@med.univ-tours.fr; hderocquigny@univ-tours.fr

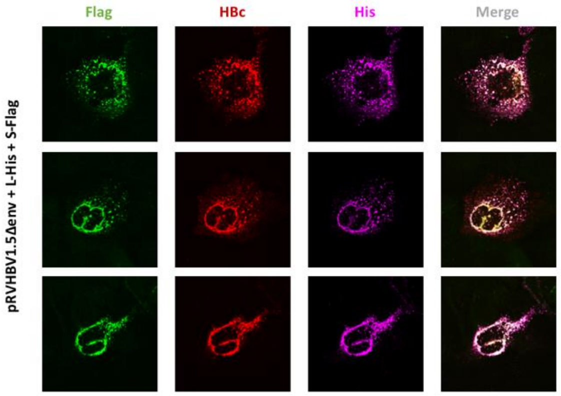

Figure S3. Detection of the tripartite complex formed by the core, L and S proteins in HBV-replicating cells. Huh7 cells were transfected with a plasmid pRVHBV1.5Δenv derived from the pRVHBV1.5 and allowing the replication of the HBV genome but defective for the expression of envelope protein as previously described[26]. Coexpression of envelope proteins was done by cotransfection with plasmids coding for S-Flag and L-His proteins. Three days after transfection,

cells were fixed and stained with anti-Flag (in green), anti-HBc (in red) and anti-His (in pink) antibodies. The three rows correspond to three different transfected cells.
