# Supplementary material for: Direct interaction between the hepatitis B virus core and envelope proteins analyzed in a cellular context
Source: Sci Rep. 2019 Nov 7;9:16178. doi: 10.1038/s41598-019-52824-z (PMC6838148; doi:10.1038/s41598-019-52824-z)
Supplement: Supplementary file 4 — Figure_S4 [file 41598_2019_52824_MOESM4_ESM.pdf]

Direct interaction between the hepatitis B virus core and envelope proteins analyzed in a cellular context

Florentin Pastor[1], Charline Herrscher[1], Romuald Patient[1], Sebastien Eymieux[1], Alain Moreau[1], Julien Burlaud-Gaillard[2], Florian Seigneuret[1], Hugues de Rocquigny[1,*], Philippe Roingeard[1,2*] and Christophe Hourioux[1,2,*]

[1]: INSERM U1259 MAVIVH – University of Tours and CHRU of Tours, Tours, France

[2]: Plate-Forme IBiSA des Microscopies, PPF ASB – University of Tours and CHRU of Tours, Tours, France.

To whom correspondence should be addressed:

[*]: hourioux@med.univ-tours.fr ; roingeard@med.univ-tours.fr; hderocquigny@univ-tours.fr

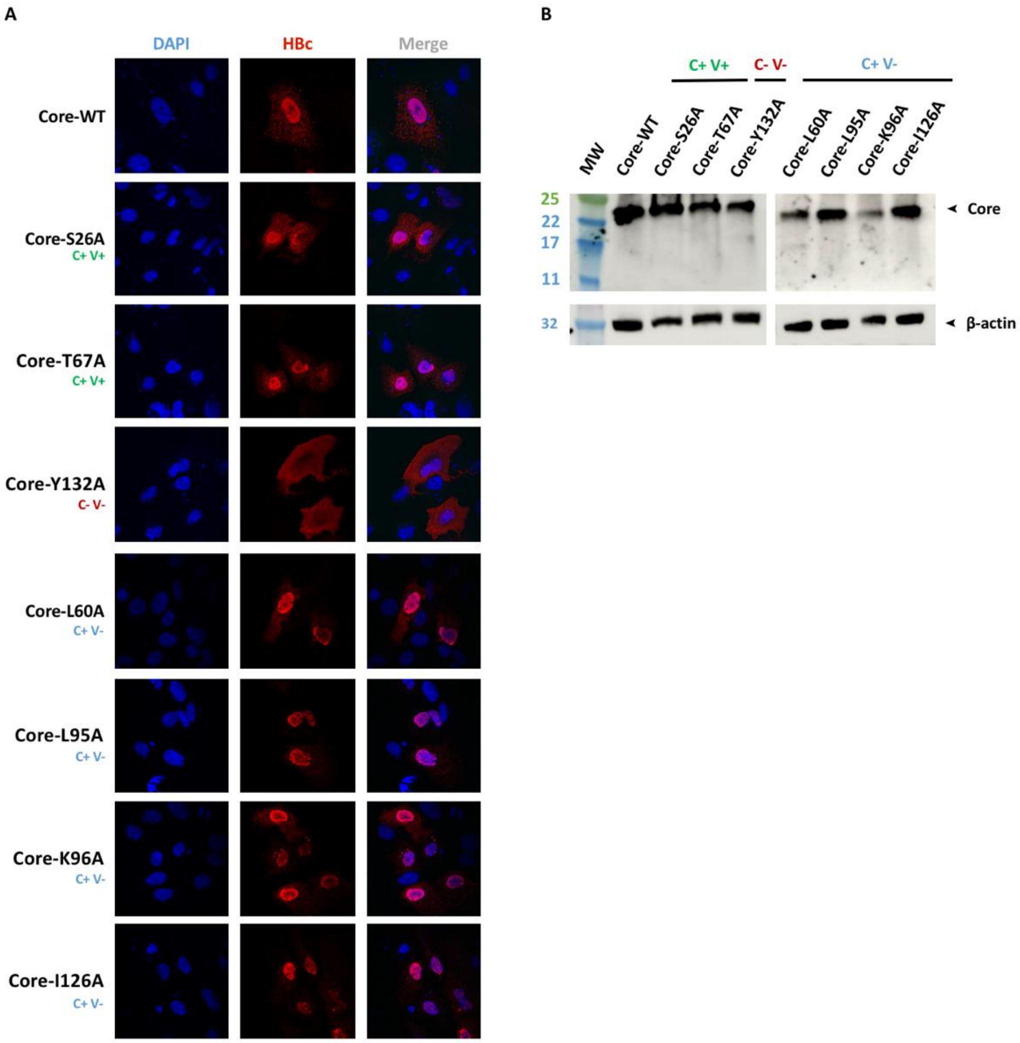

Figure S4. Expression of the MBD-mutant core proteins. Huh7 cells were transfected by incubation for three days with plasmids encoding the WT core protein or one of the seven mutant core proteins. **(A)** The cellular distribution of each of the encoded proteins was evaluated by confocal immunofluorescence microscopy after the staining of the cells with an anti-HBc antibody (in red) and counterstaining with DAPI (in blue). **(B)** Cell lysates were also analyzed by western blotting with an anti-HBc antibody, with β-actin levels used for normalization. The capsid formation and virion secretion abilities of each mutant protein are reported at the top of the figure.
