# Supplementary material for: Direct interaction between the hepatitis B virus core and envelope proteins analyzed in a cellular context
Source: Sci Rep. 2019 Nov 7;9:16178. doi: 10.1038/s41598-019-52824-z (PMC6838148; doi:10.1038/s41598-019-52824-z)
Supplement: Supplementary file 5 — Supplemental_Table_1 [file 41598_2019_52824_MOESM5_ESM.pdf]

Direct interaction between the hepatitis B virus core and envelope proteins analyzed in a cellular context

Florentin Pastor[1], Charline Herrscher[1], Romuald Patient[1], Sebastien Eymieux[1], Alain Moreau[1], Julien Burlaud-Gaillard[2], Florian Seigneuret[1], Hugues de Rocquigny[1,*], Philippe Roingeard[1,2*] and Christophe Hourioux[1,2,*]

[1]: INSERM U1259 MAVIVH – University of Tours and CHRU of Tours, Tours, France

[2]: Plate-Forme IBiSA des Microscopies, PPF ASB – University of Tours and CHRU of Tours, Tours, France.

To whom correspondence should be addressed:

[*]: hourioux@med.univ-tours.fr ; roingeard@med.univ-tours.fr; hderocquigny@univ-tours.fr

Amino acids	Genotypes (number of analyzed sequences)							
	A (1417)	B (2648)	C (2638)	D (1724)	E (482)	F (250)	G (86)	H (34)
S26	99.4	95.5	98.1	98.9	99.8	99.6	100.0	100.0
L60	98.0	83.4	96.4	99.0	98.1	97.6	98.8	94.1
T67	96.6	93.8	97.2	88.5	87.8	96.8	100.0	97.1
L95	98.6	95.4	98.1	99.1	99.0	100.0	100.0	100.0
K96	99.4	99.3	99.6	99.9	99.4	100.0	100.0	100.0
I126	99.9	99.7	99.7	99.8	100.0	100.0	100.0	100.0
Y132	99.9	100.0	100.0	99.7	100.0	100.0	100.0	100.0

Supplemental Table 1. Conservation percentage of residues used in our study in all HBV genotype. All HBV sequences were obtained from the HBV database at IBCP (Lyon, France, https://hbvdb.ibcp.fr/HBVdb/)[53]. We chose to obtain all the complete HBV core proteins sequences available, constituting a potentially more biological relevant source of functional circulating

viruses. The sequences were first aligned using clustalX software (http://www.clustal.org/clustal2/). After data curating, conservation percentages of amino-acids were calculated for each genotype. N-terminal part of genotype G core sequences carry an extra 12 amino-acid sequence insertion[54], which was taken into account to determine the corresponding amino acid positions.
