# Supplementary material for: Direct interaction between the hepatitis B virus core and envelope proteins analyzed in a cellular context
Source: Sci Rep. 2019 Nov 7;9:16178. doi: 10.1038/s41598-019-52824-z (PMC6838148; doi:10.1038/s41598-019-52824-z)
Supplement: Supplementary file 6 — Supplemental_Table_2 [file 41598_2019_52824_MOESM6_ESM.pdf]

Direct interaction between the hepatitis B virus core and envelope proteins analyzed in a cellular context

Florentin Pastor[1], Charline Herrscher[1], Romuald Patient[1], Sebastien Eymieux[1], Alain Moreau[1], Julien Burlaud-Gaillard[2], Florian Seigneuret[1], Hugues de Rocquigny[1,*], Philippe Roingeard[1,2*] and Christophe Hourioux[1,2,*]

[1]: INSERM U1259 MAVIVH – University of Tours and CHRU of Tours, Tours, France

[2]: Plate-Forme IBiSA des Microscopies, PPF ASB – University of Tours and CHRU of Tours, Tours, France.

To whom correspondence should be addressed:

[*]: hourioux@med.univ-tours.fr ; roingeard@med.univ-tours.fr; hderocquigny@univ-tours.fr

Plasmid name	Primer sequence
**Envelope proteins constructions**	
L-His	Fwd : 5’ TT**A-AGC-TT**A-TGG-GAG-GTT-GGT-CAT-CA 3’
	Rev : 5’ GA**C-TCG-AG**T-TAG-TGG-TGA-TGG-TGA-TGA-TGA-GCA-TAA-TCA-GGA-ACA-TCA-TAA-GG 3’
L	Fwd : 5’ AA**A-AGC-TT**A-TGG-GAG-GTT-GGT-CAT-CA 3’
	Rev : 5’ TT**C-TCG-AG**T-TAA-ATG-TAT-ACC-CAG-AGA-CAA-AAG-AAA-ATT-GG 3’
L-His-Δ1	Fwd : 5’ CAA-GGC-TCA-CCC-CTC-CAC-ACA-ATC-GGC-AGT-CAG-GAA-GGC-AGC 3’
	Rev : 5’ GCC-TTC-CTG-ACT-GCC-GAT-TGT-GTG-GAG-GGG-TGA-GCC-TTG-G 3’
L-His-Δ2	Fwd : 5’ TCC-TCC-TCC-TGC-CTC-CAC-CAC-TGC-CTT-CCA-CCA-AAC-TC 3’
	Rev : 5’ GAG-TTT-GGT-GGA-AGG-CAG-TGG-TGG-AGG-CAG-GAG-GAG 3’
L-His-Δ3	Fwd : 5’ GGC-CAT-GCA-GTG-GAA-TTC-CGG-AAC-AGT-AAA-CCC-TGC-TCC 3’
	Rev : 5’ GAG-CAG-GGT-TTA-CTG-TTC-CGG-AAT-TCC-ACT-GCA-TGG-CCT-G 3’
S-His	Fwd : 5’ TT**A-AGC-TT**A-TGG-AGA-ACA-TCA-CAT-CAG-GAT-TC 3’
	Rev : 5’ GA**C-TCG-AG**T-TAG-TGG-TGA-TGG-TGA-TGA-TGA-GCA-TAA-TCA-GGA-ACA-TCA-TAA-GG 3’
S	Fwd : 5’ AA**A-AGC-TT**A-TGG-AGA-ACA-TCA-CAT-CAG-GAT-TC 3’
	Rev : 5’ TT**C-TCG-AG**T-TAA-ATG-TAT-ACC-CAG-AGA-CAA-AAG-AAA-ATT-GG 3’
**Core protein constructions**	
Core	Fwd : 5’ AA**C-TTA-AG**G-CGG-CCG-CCG-CCA-CCA-TGG-ATA-TCG-ATC-C 3’
	Rev : 5’ AA**G-GGC-CC**G-CTA-GCT-TCA-CTA-ACA-TTG-AGA-TTC-CCG-A 3’
Core-S26A	Fwd : 5’ CTT-CTT-TCC-TGC-CGT-ACG-AGA-CCT-TC 3’
	Rev : 5’ TCA-CTC-GGG-AGA-AAC-GAG 3’
Core-L60A	Fwd : 5’ ATT-GCC-TGC-TGG-GGG-GAG-CTC 3’
	Rev : 5’ TGC-TTG-CCT-GAG-GGC-AGT-ATG 3’,
Core-T67A	Fwd : 5’ GGA-GCT-CAT-GGC-CCT-GGC-CAC-GT 3’
	Rev : 5’ CCC-CAG-CAA-AGA-ATT-GCT-TGC 3’
Core-L95A	Fwd : 5’ TAA-TAT-GGG-TGC-CAA-GTT-CAG-GCA-AC 3’
	Rev : 5’ GTG-TTG-ACA-TAA-CTG-ACT-AC 3’
Core-K96A	Fwd : 5’ TAT-GGG-TTT-AGC-CTT-CAG-GCA-ACT-CTT-GTG 3’
	Rev : 5’ TTA-GTG-TTG-ACA-TAA-CTG-AC 3’
Core-I126A	Fwd : 5’ AGT-GTG-GGC-CAG-AAC-TCC-TC 3’
	Rev : 5’ CCG-AAA-GAC-ACC-AAA-TAT-TCT-ATA-ACT-G 3’
Core-Y132A	Fwd : 5’ TCC-TCC-AGC-TGC-CAG-GCC-TCC-GA 3’
	Rev : 5’ GTT-CTG-ATC-CAC-ACT-CCG 3’

Supplemental Table 2. Primers used for polymerase chain reactions. The forward (Fwd) and reverse (Rev) primers for the WT S and L constructs contain *Hind*III and *Xho*I restriction sites (in bold, AAGCTT and CTCGAG, respectively). They were used for the generation of all envelope protein constructs from pcDNA3.1(+). The primers for the L-Δ1-His, L-Δ2-His and L-Δ3-His mutants deleted the chosen sequence (72-97, 98-124 or 125-148 residues,

respectively). The primers for the WT core protein contain *Afl*II and *Apa*I restriction sites (in bold, CTTAAG and GGGCCC, respectively) for cloning in pCDNA3.1(+). The primers for the core-S26A, -L60A, -T67A, -L95A, -K96A, -I126A and -Y132A mutants replaced the chosen amino-acid residue with an alanine.
