# Supplementary material for: Direct interaction between the hepatitis B virus core and envelope proteins analyzed in a cellular context
Source: Sci Rep. 2019 Nov 7;9:16178. doi: 10.1038/s41598-019-52824-z (PMC6838148; doi:10.1038/s41598-019-52824-z)
Supplement: Supplementary file 7 — Supplemental_information_raw_data [file 41598_2019_52824_MOESM7_ESM.pdf]

Tittle Page

Direct interaction between the hepatitis B virus core and envelope proteins analyzed in a cellular context

Authors

Florentin Pastor[1], Charline Herrscher[1], Romuald Patient[1], Sebastien Eymieux[1], Alain Moreau[1], Julien Burlaud-Gaillard[2], Florian Seigneuret[1], Hugues de Rocquigny[1,*], Philippe Roingeard[1,2*] and Christophe Hourioux[1,2,*]

[1]: INSERM U1259 MAVIVH – University of Tours and CHRU of Tours, Tours, France

[2]: Plate-Forme IBiSA des Microscopies, PPF ASB – University of Tours and CHRU of Tours, Tours, France.

To whom correspondence should be addressed:

[*]: hourioux@med.univ-tours.fr ; roingeard@med.univ-tours.fr; hderocquigny@univ-tours.fr

To whom it may concern:

This document provides information on the raw blots used to generate the final figures associated with the submitted manuscript. These blots are shown as raw data, according to the request of Scientific reports manuscript admin. None of these blots was recorded using multiple exposures or was manipulated using an image processing software.

This document aims also to synthesize all the modifications needed to finalize the paper submission. For each figure, we provide:

- images of the raw blots, with its corresponding filename used for uploading ;
- a specific comment explaining modifications done to generate the final figure.

Yours sincerely,

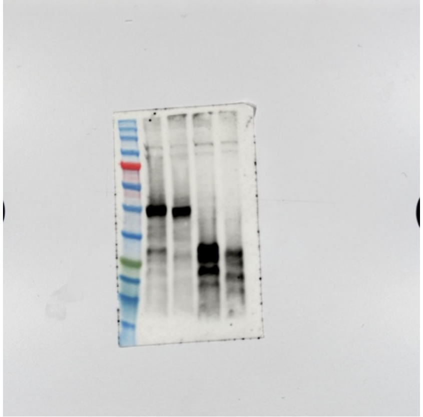

Fig_2A1_raw

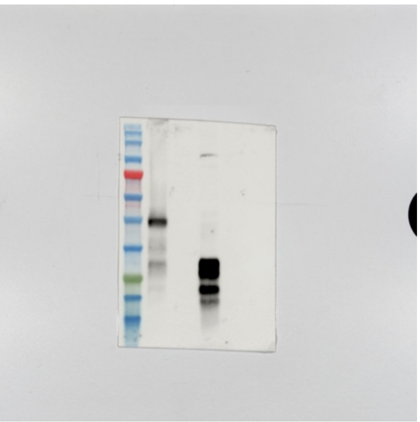

Fig_2A2_raw

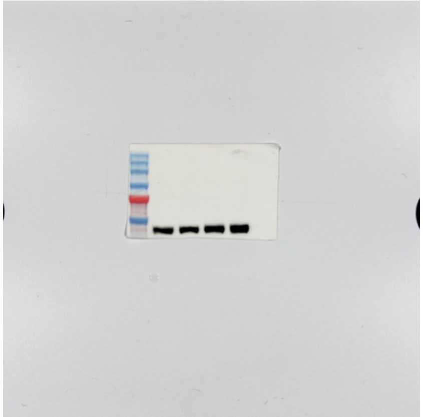

Fig_2A3_raw

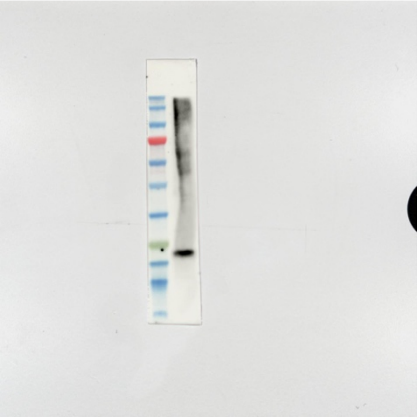

Fig_2A4_raw

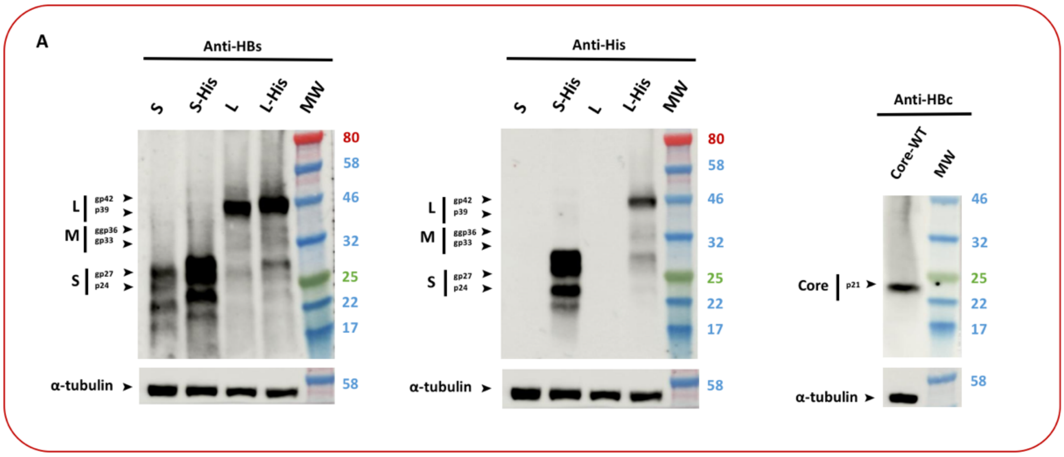

Figure 2. Envelope and core protein levels. Blots were cropped from raw files. Note that all blots were horizontally mirrored in the definitive submitted figure.

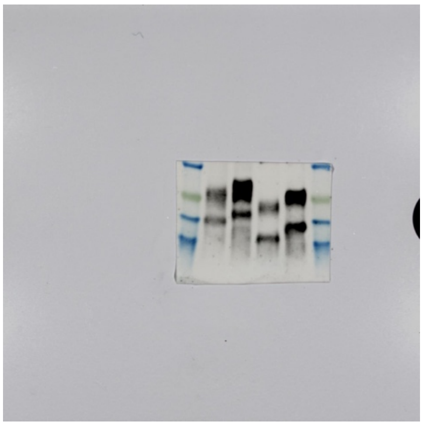

Fig_S1.1_raw

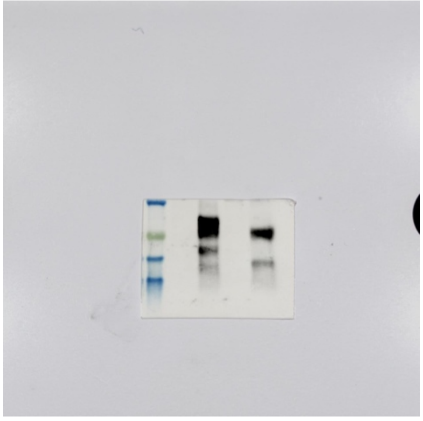

Fig_S1.2_raw

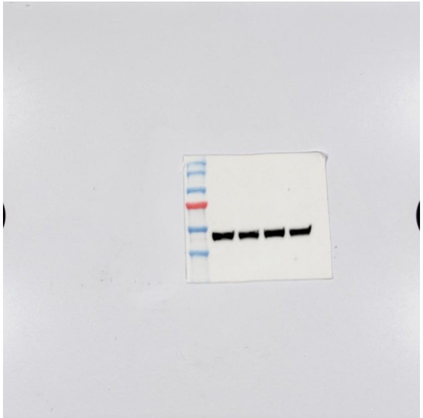

Fig_S1.3_raw

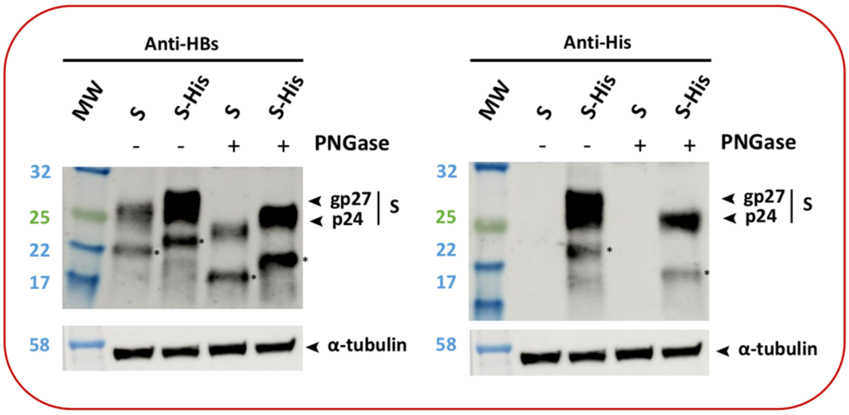

Figure S1. PNGase treatment of S and S-His proteins. Blots were cropped from raw files.

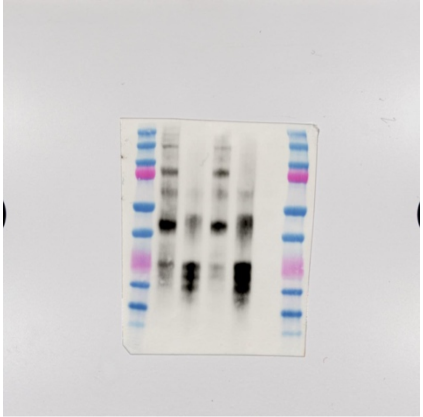

Fig_3C1_raw

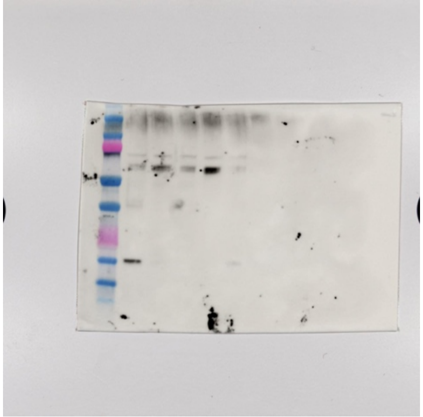

Fig_3C2_raw

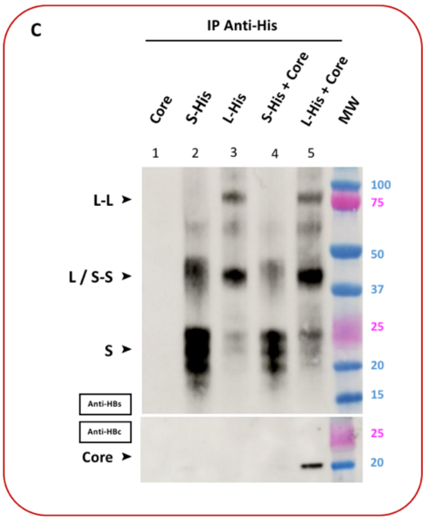

Figure 3. Only the L protein interacts with the core protein. Extra lanes and supplemental molecular weight markers were cropped. Blots were horizontally mirrored in the submitted figure.

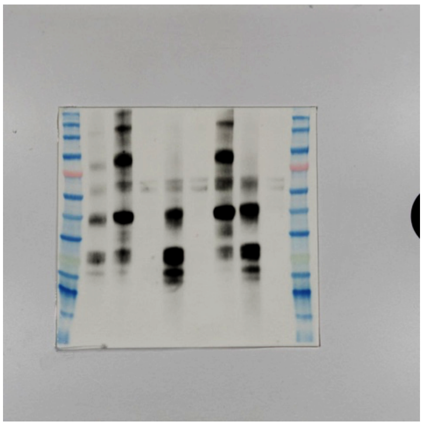

Fig_4C1_raw

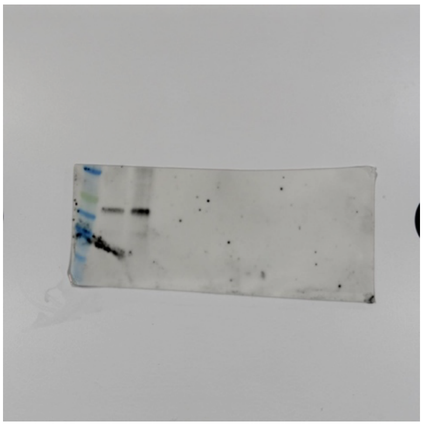

Fig_4C2_raw

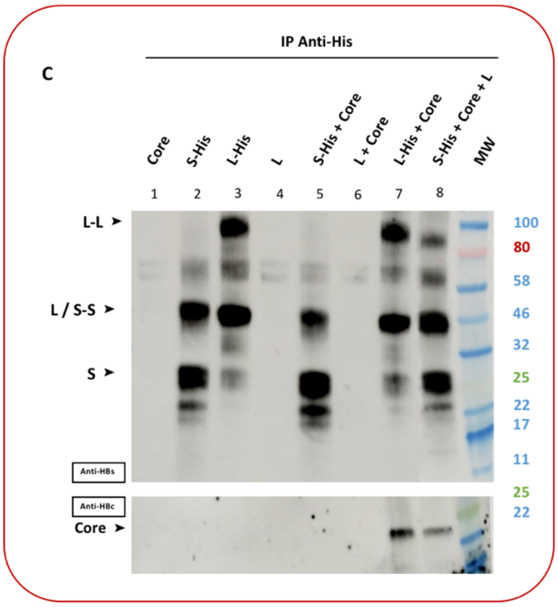

Figure 4. Tripartite interaction between the L, S and core proteins. As recommended in the digital image and integrity policies, cropped gels were replaced by the raw blot for IP and co-IP. An additional band (lane 4) is present in the new submitted figure, corresponding to a redundant control which was initially cropped. The corresponding new lanes numbers were modified in the manuscript according to the modifications introduced in this modified Figure 4. The new version was uploaded to replace the initial one.

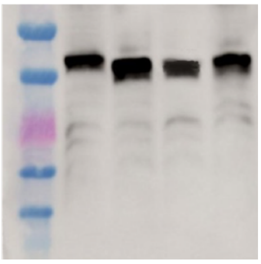

Fig_S2.1_raw

Figure S2. Expression of the mutant L proteins. Raw filenames are presented under each blot.

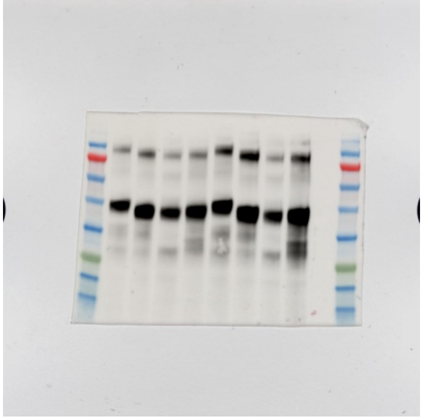

Fig_5C1_raw

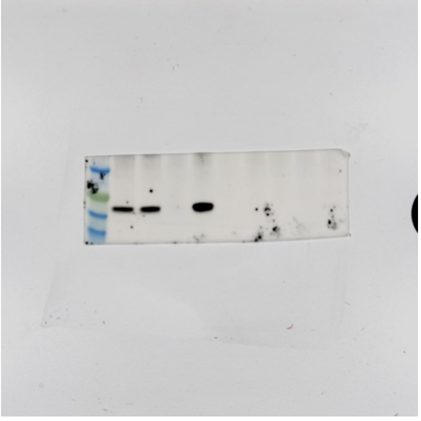

Fig_5C2_raw

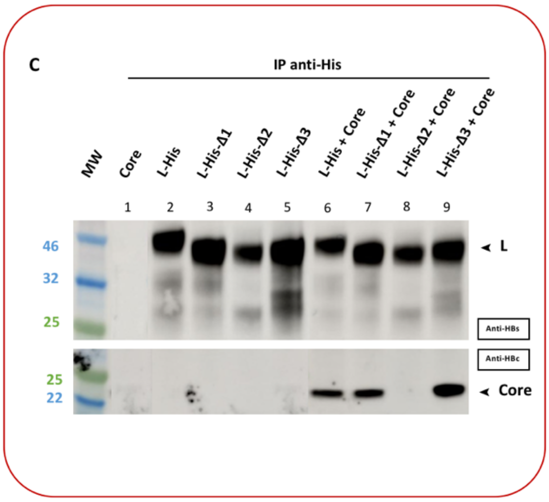

Figure 5. Interaction between the mutant L proteins and the core protein. Blots were horizontally flipped and cropped to remove dimeric proteins bands of high molecular weight (as mentioned in the manuscript) and the supplemental molecular weight markers.

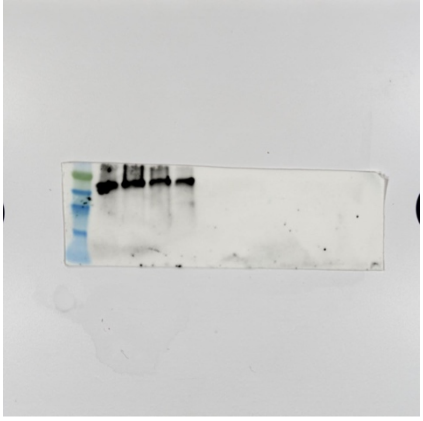

Fig_S4.1_raw

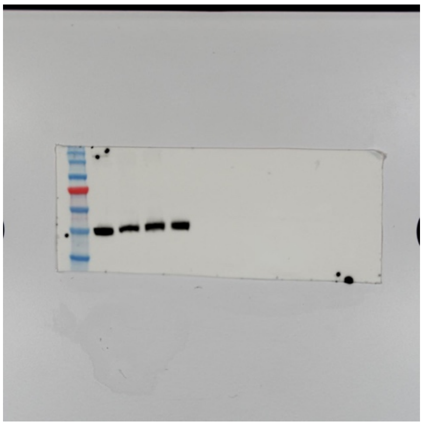

Fig_S4.2_raw

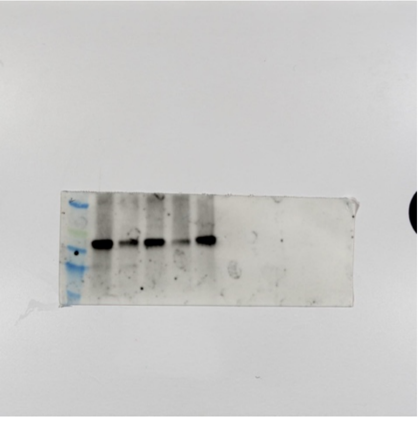

Fig_S4.3_raw

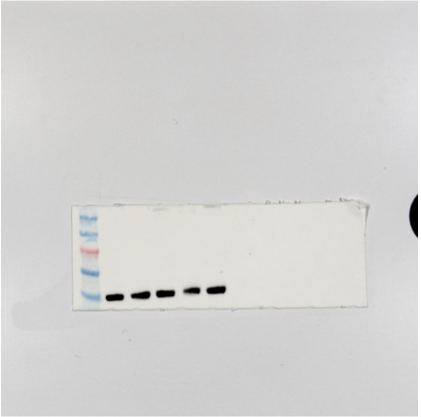

Fig_S4.4_raw

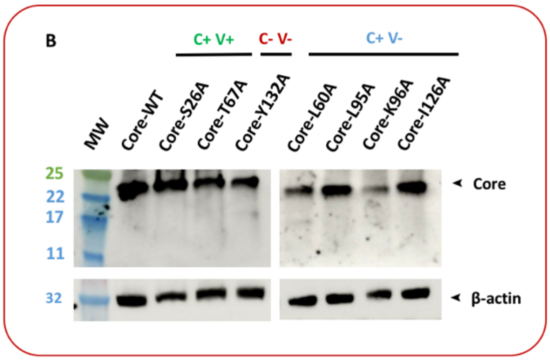

Figure S4. Expression of the MBD-mutant core proteins. Blots were cropped to remove unused gel lanes.

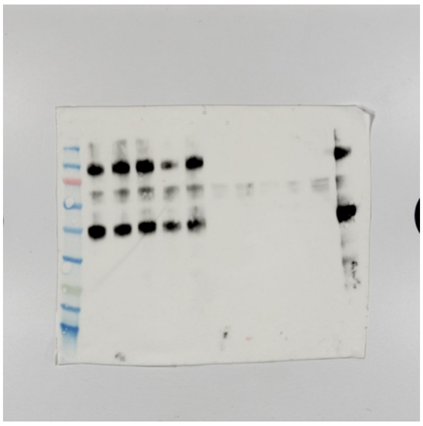

Fig_6C1_raw

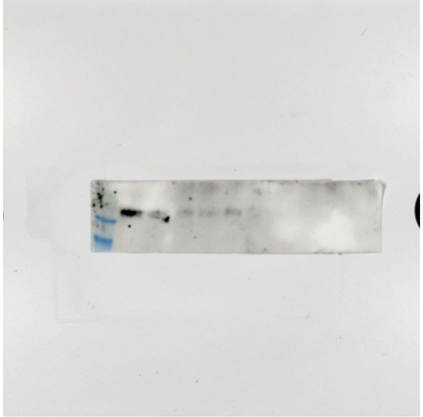

Fig_6C2_raw

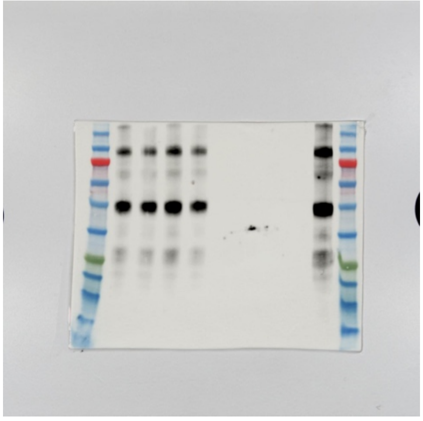

Fig_6C3_raw

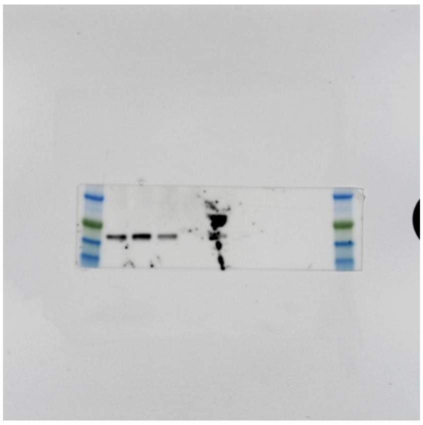

Fig_6C4_raw

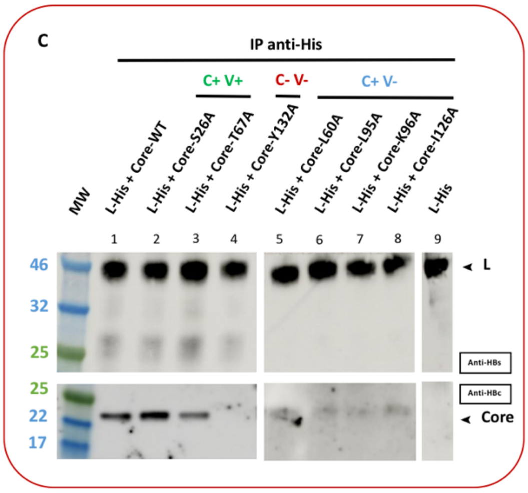

Figure 6. Interaction between the L protein and the mutant core proteins. All blot acquisitions were processed in parallel and correspond to the same experiment. This comment was added into the figure legend. Blots were cropped to remove additional non relevant control and additional molecular weight markers.
